# Antinociceptive effects of incarvillateine, a monoterpene alkaloid from *Incarvillea sinensis*, and possible involvement of the adenosine system

**DOI:** 10.1038/srep16107

**Published:** 2015-11-03

**Authors:** Mei-Liang Wang, Gang Yu, Shou-Pu Yi, Feng-Ying Zhang, Zhi-Tong Wang, Bin Huang, Rui-Bin Su, Yan-Xing Jia, Ze-Hui Gong

**Affiliations:** 1 State Key Laboratory of Toxicology and Medical Countermeasures, Beijing Key Laboratory of Neuropsychopharmacology, Beijing Institute of Pharmacology and Toxicology, 27 Taiping Road, Beijing 100850, China; 2 State Key Laboratory of Natural and Biomimetic Drugs, School of Pharmaceutical Sciences, Peking University, 38 Xueyuan Road, Beijing 100191, China

## Abstract

*Incarvillea sinensis* is a Bignoniaceae plant used to treat rheumatism and relieve pain in traditional Chinese medicine. As a major component of *I. sinensis*, incarvillateine has shown analgesic activity in mice formalin tests. Using a series of animal models, this study further evaluated the effects of incarvillateine against acute, inflammatory, and neuropathic pain. Incarvillateine (10 or 20 mg/kg, i.p.) dose-dependently attenuated acetic acid-induced writhing, but did not affect thermal threshold in the hot plate test. In a Complete Freund’s Adjuvant model, incarvillateine inhibited both thermal hyperalgesia and paw edema, and increased interleukin-1β levels. Additionally, incarvillateine attenuated mechanical allodynia induced by spared nerve injury or paclitaxel, whereas normal mechanical sensation was not affected. Incarvillateine did not affect locomotor activity and time on the rotarod at analgesic doses, and no tolerance was observed after 7 consecutive daily doses. Moreover, incarvillateine-induced antinociception was attenuated by theophylline, 1,3-dipropyl-8-cyclopentylxanthine, and 3,7-dimethyl-1-propargylxanthine, but not naloxone, indicating that the effects of incarvillateine on chronic pain were related to the adenosine system, but not opioid system. These results indicate that incarvillateine is a novel analgesic compound that is effective against inflammatory and neuropathic pain, and that its effects are associated with activation of the adenosine system.

Pain is the most common reason people seek medical care, and treatment of pain remains challenging in the clinic. In particular, chronic inflammatory and neuropathic pain can persist and significantly affect the health and quality of life of patients. Various drug classes, including antidepressants, anti-epileptics, opioids, and topical anesthetics, are used to treat pain in the clinic^1^. However, their clinical applications are limited by either poor therapeutic action and/or marked side effects^2^. Therefore, there is a constant demand for novel compounds that will effectively inhibit pathological pain with limited influence on normal nociception and other physiological functions.

Among various therapeutic targets, the adenosine receptor system is promising for the treatment of pain^3^. Adenosine receptors, namely A_1_, A_2_A, A_2_B, and A_3_ receptors, are widely distributed in the spinal cord and brain areas involved in pain transmission, as well as peripheral sensory afferents or adjacent cells^4^,^5^,^6^. Administration of adenosine or adenosine receptor agonists inhibits pain-related behaviors in animal and human models^7^,^8^,^9^,^10^. Compared to classical analgesics, such as opioids, adenosine receptor agonists are particularly effective in chronic pain that arises from inflammation and neuropathy^11^,^12^. However, direct-acting agonists may exert CNS side-effects, including general sedation and locomotor suppression, which raises serious concerns when considering the development of agents affecting the adenosine system^13^,^14^.

*Incarvillea sinensis* is a Bignoniaceae plant that has been widely used as a herbal medicine for more than 1400 years in China. In traditional Chinese medicine, *I. sinensis* is used to treat rheumatism, bruises, and wounds, and is effective in attenuating pain and inflammation^15^. Since the 1990 s, photochemists and pharmacologists have investigated the active components in *I. sinensis*, isolating more than 13 monoterpene alkaloids and 7 macrocyclic spermine alkaloids^16^,^17^,^18^,^19^,^20^,^21^,^22^,^23^,^24^. Incarvillateine (Fig. 1) is considered the major active component, with a characteristic dimeric structure and five contiguous stereocenters on the bicyclic piperidine moiety^16^. Previous reports showed that incarvillateine attenuates formalin-induced pain in mice with a higher potency than morphine^25^. Moreover, the antinociceptive effect of incarvillateine in this model can be attenuated by non-selective adenosine receptor antagonist, non-selective opioid receptor antagonist, and μ and κ opioid receptor antagonists^25^,^26^. Nevertheless, no other study has addressed the pharmacological effects of incarvillateine. The difficult isolation procedure and small quantity yield also pose hurdles for further research and development of incarvillateine.

For more than a decade, efforts have been made to artificially synthesize incarvillateine, and its total synthesis was achieved in 2004^27^,^28^,^29^. We also developed a method for concise, total, enantioselective synthesis of incarvillateine in a rapid, efficient, and economic manner^30^. Taking advantage of these improvements, we investigated the pharmacological effects of incarvillateine in a set of experimental pain models, with a focus on its effects on chronic pathological pain. Furthermore, we explored possible mechanisms underlying its antinociceptive effects. Our results showed that incarvillateine is a novel analgesic for inflammatory and neuropathic pain, with limited influence on normal pain and motor function. The effects of incarvillateine against pathological pain may be closely related to the activation of the adenosine receptor system, but not the opioid receptor system.

## Results

### Effect of incarvillateine in the writhing and hot plate tests

**Figure 2:**
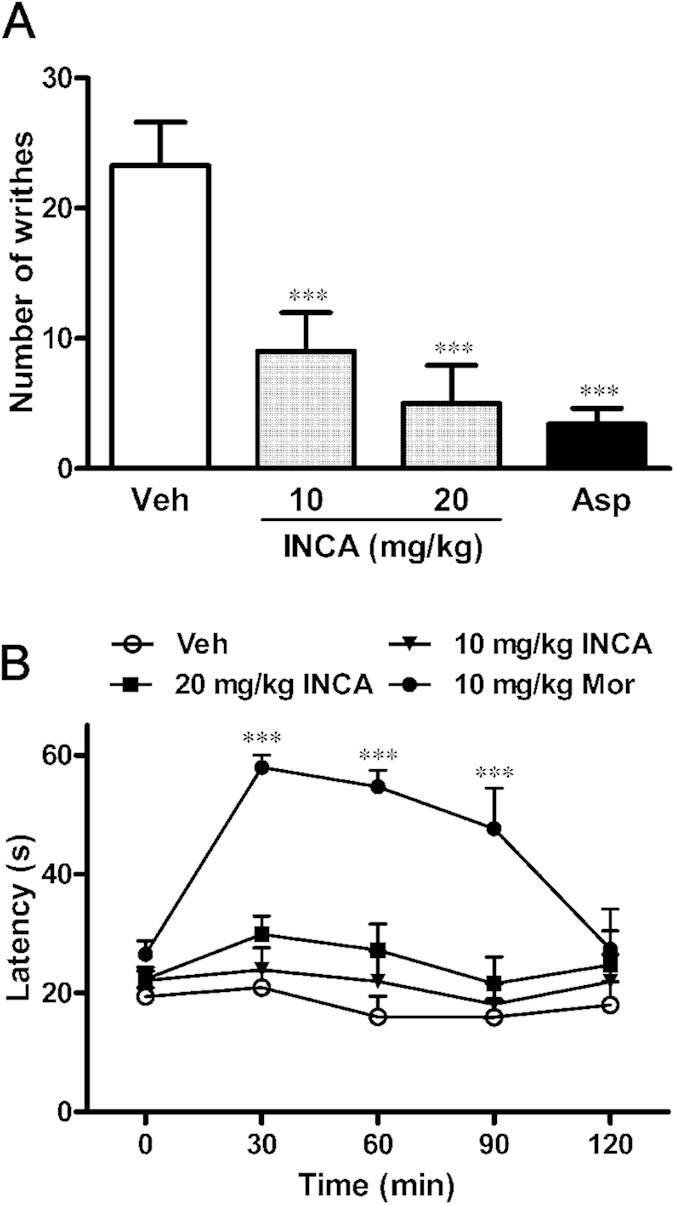
Effect of incarvillateine on pain behaviors in the mice acetic acid writhing (**A**) and hot plate (**B**) tests. In the writhing test, mice were intraperitoneally administered vehicle (Veh), incarvillateine (INCA, 10 or 20 mg/kg), or aspirin DL-lysine (Asp, 100 mg/kg) 30 min prior to acetic acid injection. In the hot plate test, mice were intraperitoneally administered vehicle (Veh), incarvillateine (INCA, 10 or 20 mg/kg), or subcutaneously administered morphine (Mor, 10 mg/kg), and tested before and at different times after drug administration. Each data point represents the mean ± S.E.M., n = 10. ***P < 0.001, compared with vehicle-treated mice.

The antinociceptive effects of incarvillateine on acute pain were evaluated using the mice writhing and hot plate tests. In the writhing test, intraperitoneal (i.p.) injection of acetic acid induced significant nociceptive responses in vehicle-treated mice. Administration of incarvillateine 30 min before acetic acid injection resulted in a dose-dependent antinociceptive effect [*F*(3, 35) = 11.33, *p* < 0.001], producing 61.4 and 78.5% inhibition of the pain behavior at doses of 10 and 20 mg/kg, respectively. The classical NSAID, aspirin DL-lysine (100 mg/kg, i.p.), also produced 85.4% inhibition of pain (Fig. 2A). In the 55 °C hot plate test, incarvillateine did not affect the pain threshold significantly, whereas morphine (10 mg/kg, s.c.) produced a potent antinociceptive effect up to 90 min after administration (Fig. 2B).

### Incarvillateine reduced Complete Freund’s adjuvant (CFA)-induced inflammation and pain

**Figure 3:**
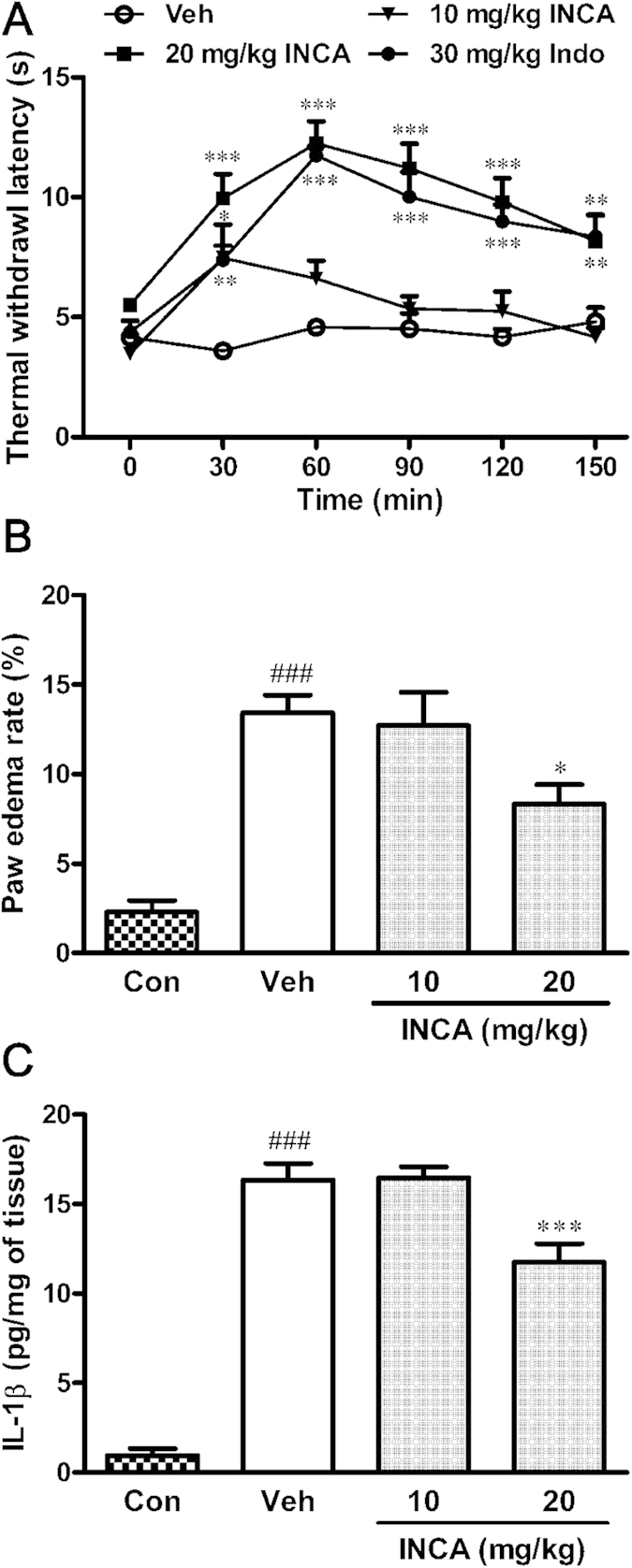
Effects of incarvillateine on CFA-induced inflammation and pain. (**A**) At 48 h after CFA injection, mice were intraperitoneally administered vehicle (Veh), incarvillateine (INCA, 10 or 20 mg/kg), or indomethacin (Indo, 30 mg/kg), and the thermal withdrawal latency was measured before and at different times after drug administration. (**B**) Paw edema was measured 30 min after intraperitoneal administration of vehicle (Veh) and incarvillateine (INCA, 10, 20 mg/kg). (**C**) IL-1β levels in inflamed hindpaw tissue were determined by enzyme-linked immunosorbent assay (ELISA) immediately after paw edema measurements. Each data point represents the mean ± S.E.M., n = 7–13. *P < 0.05, **P < 0.01, ***P < 0.001, compared with vehicle-treated mice. ^###^*P* < 0.001, compared with control mice that received saline instead of CFA.

Injection of CFA in the paw is an established model of inflammation that induces thermal hyperalgesia from 24 to 72 h^31^. Consistent with previous reports, thermal withdrawal latency was decreased 48 h after CFA injection. Administration of incarvillateine dose-dependently attenuated CFA-induced thermal hyperalgesia [*F*(3, 140) = 51.71, *p* < 0.001], with significant effects observed at both 10 and 20 mg/kg 30 min after administration. At 20 mg/kg, the effect of incarvillateine lasted for more than 150 min. Indomethacin, a positive control, also significantly attenuated CFA-induced decreases in withdrawal latency (Fig. 3A).

In addition to attenuating thermal hyperalgesia, incarvillateine also attenuated CFA-induced paw edema. As shown in Fig. 3B, intraplantar injection of CFA resulted in a significant increase in paw thickness. Incarvillateine (20 mg/kg) produced a mild, but significant, inhibition of paw edema [*F*(3,45) = 16.91, *p* < 0.001]. Consistent with this observation, the CFA-induced increase in tissue interleukin (IL)-1β was significantly inhibited by 20 mg/kg incarvillateine [*F*(3,27) = 89.06, *p* < 0.001; Fig. 3C].

### Incarvillateine reduced spared nerve injury (SNI)-induced neuropathic pain

**Figure 4:**
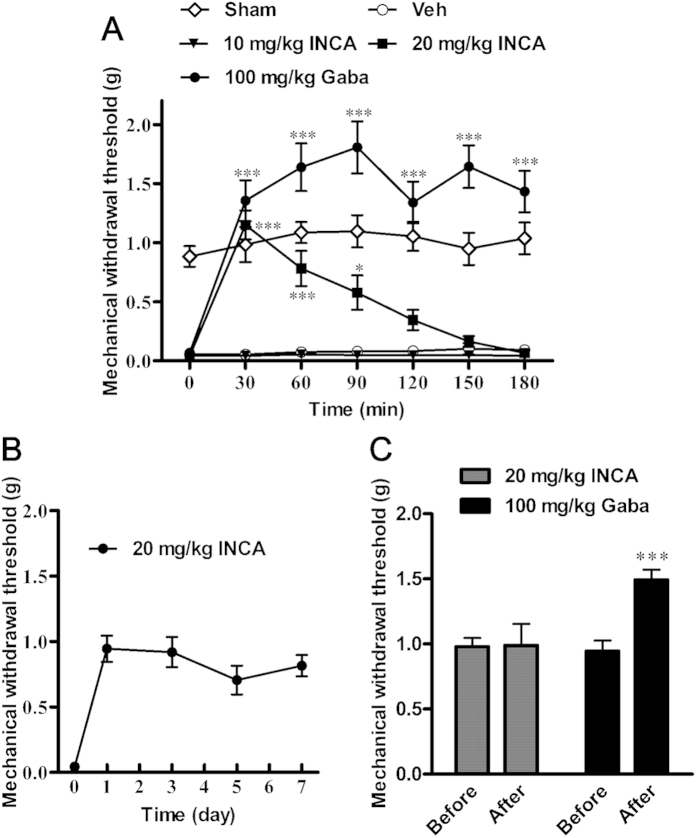
Effects of incarvillateine on SNI-induced neuropathic pain. (**A**) After 5–7 days of recovery from SNI surgery, mice were intraperitoneally administered vehicle (Veh), incarvillateine (INCA, 10 or 20 mg/kg), or gabapentin (Gaba, 100 mg/kg). The mechanical withdrawal threshold was measured using von Frey filaments before and at different times after drug administration. (**B**) Mice were intraperitoneally administered with incarvillateine (INCA, 20 mg/kg) once daily for 7 consecutive days and the mechanical threshold was measured 30 min after administration on day 1, 3, 5, and 7. (**C**) Control mice naïve to SNI injury were intraperitoneally administered incarvillateine (INCA, 20 mg/kg) or gabapentin (Gaba, 100 mg/kg), and the mechanical threshold was measured 30 min after administration. Each data point represents the mean ± S.E.M., n = 9–10. *P < 0.05, ***P < 0.001, compared with vehicle-treated mice.

As shown in Fig. 4A, SNI surgery, which reflects neurotrauma in the clinic, induced significant mechanical allodynia. Compared to sham-operated mice, the mean mechanical sensitivity in SNI mice decreased from 0.88 to < 0.07 g. Vehicle administration did not affect the mechanical withdrawal threshold, whereas 20 mg/kg incarvillateine produced a significant antinociceptive effect for more than 90 min [*F*(4, 264) = 78.29, *p* < 0.001]. Gabapentin, a positive control, also reversed SNI-induced decreases in mechanical threshold. To investigate possible tolerance in incarvillateine-induced antinociception, a repeated dose study was conducted in the same model. As shown in Fig. 4B, no significant reduction in the antinociception was observed in SNI mice treated with 20 mg/kg incarvillateine once daily for 7 consecutive days (*p* > 0.05). Moreover, 100 mg/kg gabapentin, but not 20 mg/kg incarvillateine, affected mechanical sensitivity in normal mice (Fig. 4C).

### Incarvillateine reduced paclitaxel-induced neuropathic pain

**Figure 5:**
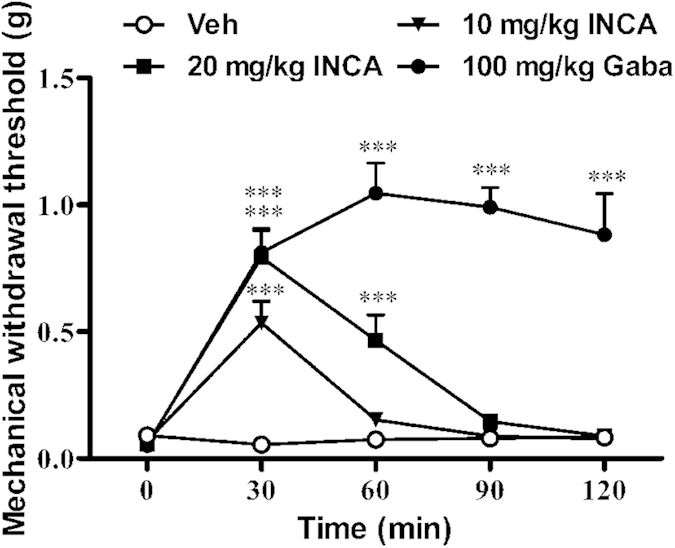
Effects of incarvillateine on paclitaxel-induced neuropathic pain. After 5 days paclitaxel treatment, mice were intraperitoneally administered vehicle (Veh), incarvillateine (INCA, 10 or 20 mg/kg), or gabapentin (Gaba, 100 mg/kg). The mechanical withdrawal threshold was measured using von Frey filaments before and at different times after drug administration. Each data point represents the mean ± S.E.M., n = 10. ***P < 0.001, compared with vehicle-treated mice.

The antiallodynic effects of incarvillateine were also investigated in paclitaxel-induced painful neuropathy, another experimental model of neuropathic pain. As expected, daily administration of 2 mg/kg paclitaxel for 5 consecutive days induced a serious decrease in mechanical withdrawal threshold, and there was no significant difference among groups before drug administration. Vehicle administration did not affect the mechanical withdrawal threshold, whereas incarvillateine produced a rapid, significant, and dose-dependent antinociceptive effect [*F*(3, 144) = 48.16, *p* < 0.001; Fig. 5]. At 20 mg/kg, the effect of incarvillateine lasted for more than 60 min. Gabapentin also reversed paclitaxel-induced decreases in mechanical threshold.

### Effects of incarvillateine on motor performance

**Figure 6:**
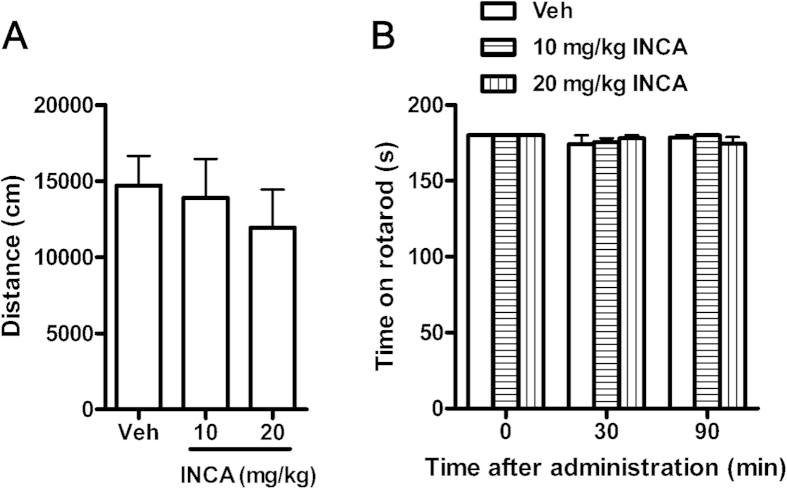
Effects of incarvillateine on motor performance. (**A**) Mice were intraperitoneally administered vehicle (Veh) or incarvillateine (INCA, 10 or 20 mg/kg), and locomotor activity was measured for 120 min. (**B**) Mice were intraperitoneally administered vehicle (Veh) or incarvillateine (INCA, 10 or 20 mg/kg), and the time on the rotarod was recorded 30 and 90 min after drug administration. Each data point represents the mean ± S.E.M., n = 10.

The possible effects of incarvillateine on motor performance were assessed by evaluating spontaneous locomotor activity and the rotarod test. Administration of 10 or 20 mg/kg incarvillateine did not affect total distance traveled [*F*(2, 27) = 0.37, *p* > 0.05; Fig. 6A] or time on the rotarod [*F*(2, 54) = 0.09, *p* > 0.05; Fig. 6B], indicating that the antinociceptive effects of incarvillateine were not due to inhibition of motor function.

### Role of opioid and adenosine receptor systems in the antinociceptive effects of incarvillateine

**Figure 7:**
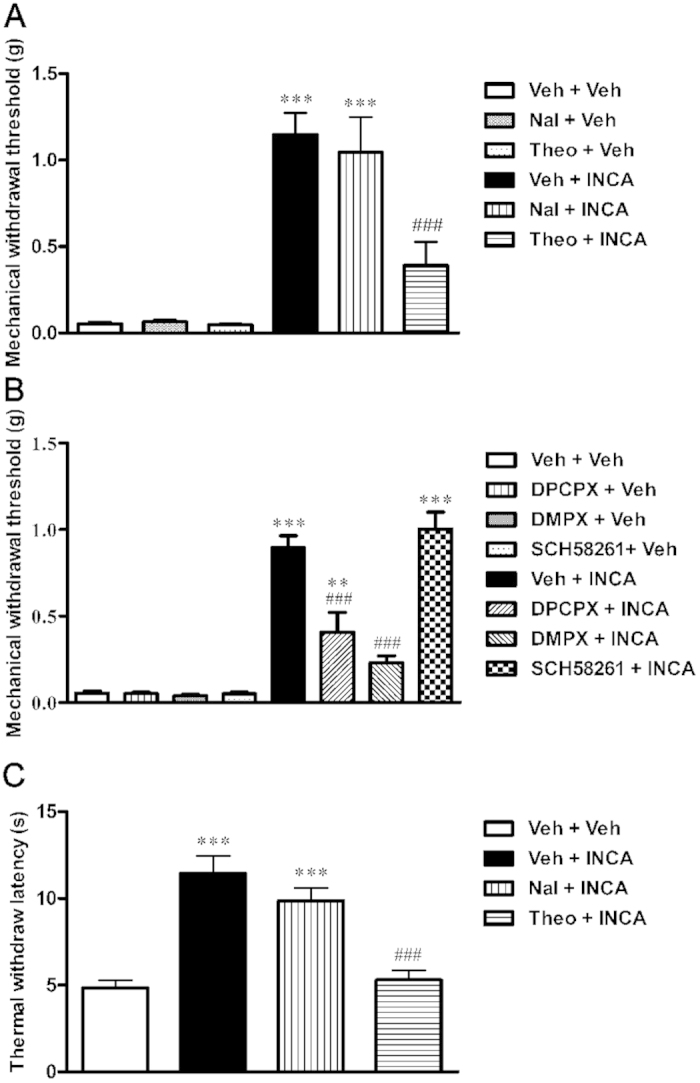
Effects of opioid and adenosine antagonists on incarvillateine-induced antinociception. (**A**) SNI mice were intracerebroventricularly administered 5 μl ACSF, the nonselective opioid antagonist naloxone (Nal, 10 nmol), or the nonselective adenosine antagonist theophylline (Theo, 10 nmol) 10 min prior to incarvillateine (INCA, 20 mg/kg) administration. The mechanical threshold was measured 30 min after incarvillateine administration. (**B**) SNI mice were intraperitoneally administered vehicle (Veh), the selective A_1_ antagonist DPCPX (0.1 mg/kg), the relatively preferential A_2_ antagonist DMPX (1 mg/kg), or the selective A_2_ _A_ antagonist SCH58261 (0.5 mg/kg) 15 min prior to incarvillateine (INCA, 20 mg/kg) administration. The mechanical threshold was measured 30 min after incarvillateine administration. (**C**) CFA mice were intracerebroventricularly administered 5 μl ACSF, the nonselective opioid antagonist naloxone (10 nmol), or the nonselective adenosine antagonist theophylline (10 nmol) 10 min prior to incarvillateine (INCA, 20 mg/kg) administration. The thermal withdrawal latency was measured 30 min after incarvillateine administration. Each data point represents the mean ± S.E.M., n = 8–10. **P < 0.01, ***P < 0.001, compared with vehicle-treated mice. ^###^*P* < 0.001, compared with incarvillateine-treated mice.

To investigate whether the antinociceptive effects of incarvillateine were mediated via activation of the opioid and/or adenosine system, the nonselective opioid antagonist naloxone, nonselective adenosine antagonist theophylline, and 3 adenosine subtype-targeting antagonists were administered before incarvillateine administration. As shown in Fig. 7A, SNI surgery induced a decrease in mechanical withdrawal threshold, with mean values less than 0.06 g. There were no significant differences among the mice intracerebroventricularly (i.c.v.) administered vehicle, naloxone, or theophylline alone. Incarvillateine produced a significant antinociceptive effect in vehicle-pretreated SNI mice 30 min after administration. Pretreatment with naloxone did not alter the effect of incarvillateine. In contrast, theophylline significantly inhibited the effect of incarvillateine [*F*(5, 52) = 19.83, *p* < 0.001], indicating that adenosine receptors may be involved in incarvillateine-induced antinociception. Furthermore, the selective A_1_ antagonist 1,3-dipropyl-8-cyclopentylxanthine (DPCPX) and the relative preferential A_2_ antagonist 3,7-dimethyl-1-propargylxanthine (DMPX), but not the selective A_2A_ antagonist SCH-58261, attenuated incarvillateine-induced antinociception [*F*(7, 62) = 40.84, *p* < 0.001; Fig. 7B].

Similar results were observed in the CFA-induced thermal pain model. Incarvillateine significantly increased the withdrawal latency in vehicle-pretreated CFA mice 30 min after administration. Pretreatment with theophylline, but not naloxone, significantly inhibited the antinociceptive effect of incarvillateine [*F*(3, 32) = 20.79, *p* < 0.001; Fig. 7C].

## Discussion

The goal of this study was to provide a systematic description of the pharmacological profile of incarvillateine, the proposed major active component of *I. sinensis*. This study extends a previous report, which evaluated incarvillateine in the formalin test, to several other pain models, including intractable inflammatory and neuropathic pain. Our results indicate that incarvillateine is a potent antiallodynic and antihyperalgesic compound, with a particular efficacy in pathological pain conditions and a mechanism involving adenosine receptor system.

The enormous clinical demands and shortage of effective therapy options has intensified research efforts to develop novel pathological pain therapies. A current research goal is to identify novel chemical structures with better or additional efficacy and/or greater safety relative to currently available treatments^32^. Active compounds from natural products and traditional herbal medicines are of interest. Incarvillateine has attracted attention due to its unique structure and potent analgesic activity in a mouse formalin model^16^,^25^. The structure of incarvillateine is dimeric, and possesses five contiguous stereocenters on the bicyclic piperidine moiety. Through analysis of basic constructive units, such as incarvilline and ferulic acid, and synthesized structure-related alkaloids, the monoterpene alkaloid and cyclobutane moieties are hypothesized to regulate the antinociceptive effects of incarvillateine. In particular, the cyclobutane ring seems indispensable, as incarvilline, incarvine C, and related chemicals lacking the cyclobutane ring showed no or weak activity^33^,^34^. To our knowledge, incarvillateine does not belong to any structural groups that have been reported to show efficacy against pathological pain.

Previous reports indicate that incarvillateine affects behavioral manifestation in the mouse formalin test, a model addressing direct nociceptor stimulation in the first phase and inflammatory pain in the second phase^35^. In this study, the effects of incarvillateine on pain perception were evaluated in detail with a series of experimental models. The hot plate test represents acute phasic thermal pain, whereas tonic pain was measured in chemical, inflammatory, and nerve-injury-induced pain models. Among these, the tonic pain models are considered to be more relevant to clinical pathological pain^36^, with each model reflecting one specific etiological factor. Incarvillateine has a broad spectrum of antinociceptive action against pathological pain, with a potency much higher than the reference drug gabapentin. However, incarvillateine did not affect acute phasic pain at the doses effective in reducing allodynia and hyperalgesia. The differential activity of incarvillateine in physiological and pathological pain may be correlated with adenosine receptor plasticity under pathological conditions^10^, and is of particular interest, as in most clinical cases the normal response to acute physiological pain is preferred while treating pathological pain. In addition, we also observed a mild anti-inflammatory effect following incarvillateine administration. Because inflammation and inflammatory mediators play an important role in pain pathology^37^, the anti-inflammatory effect, particularly the inhibition of IL-1 production, may be contributing factors in incarvillateine-induced antinociception.

To reveal the mechanism of incarvillateine in pathological pain, *in vivo* antagonist experiments were performed with specific antagonists. It was reported that the antinociceptive effects of many compounds involve activation of the opioid system by promoting endogenous peptide release or directly activating receptors, and that these effects were diminished by corresponding antagonists^38^,^39^,^40^. In this study, naloxone, a nonselective opioid antagonist, did not affect the antinociceptive effects of incarvillateine in SNI and CFA injured mice. Although the results from these two models are consistent, they appear to be in contrast to a previous report, which suggested a concomitant involvement of the opioid and adenosine receptor systems in incarvillateine-induced antinociception in the formalin test^25^. As opioid agonists are likely to be effective in the hot plate test, induce locomotor hyperactivity, and tend to develop tolerance, the lack of these characteristics in our studies did not support the involvement of the opioid receptor system in incarvillateine antinociception. Moreover, the difference in pain models used may account for the different conclusions. Nevertheless, the present results excluded the opioid mechanism of incarvillateine, at least under chronic pathological pain conditions.

Adenosine receptors, especially the A_1_ receptor, are considered attractive targets for the development of analgesics. Endogenous adenosine and selective A_1_ agonists have been well-documented to possess analgesic effects^41^. The activation of the adenosine system is involved as a mechanism not only in some analgesic compounds, but also in some therapies, such as Zusanli acupuncture, which triggers adenosine release and activates the A_1_ receptor^42^. Selective A_1_ agonists are also effective against neuropathic pain induced by SNI^7^,^43^. The attenuation of incarvillateine antinociception by the nonselective antagonist theophylline clearly suggests the involvement of the adenosine system. Further, receptor-subtype-preferential antagonists were used to distinguish whether the effects of incarvillateine were mediated through A_1_ or A_2_ receptors. The three antagonists used were DPCPX, DMPX, and SCH58261, targeting A_1_, A_2_, and A_2A_ respectively. The antinociceptive effects of incarvillateine were significantly attenuated by DPCPX and DMPX, but not SCH58261. It should be noted that, although DMPX is frequently used as an A_2A_ antagonist, its selectivity for the rat A_2A_ was only 3 fold in relative to A_1_^44^. Therefore, DMPX-induced antagonism of incarvillateine antinociception may also be due to A_1_ receptor blockade. Thus, we believe that the A_1_ receptor is the major target mediating incarvillateine antinociception. The lack of motor dysfunction also supports this hypothesis, as motor function is primarily regulated by the A_2_ receptor^45^. Although these findings underlined the involvement of the adenosine system and point to the A_1_ receptor as a mediator of incarvillateine action, further work is needed to address the specific mechanisms, including binding and activating properties and the regulatory effects of incarvillateine on different adenosine receptor subtypes, as well as its influence on endogenous adenosine release or metabolism. Moreover, the possible role of incarvillateine biotransformation cannot be neglected.

In conclusion, the present study demonstrated that incarvillateine, a monomer from *I. sinensis*, is effective against pain induced by chemical substances, inflammation, and neural injury, without any significant alterations in acute thermal and mechanical nociception, as well as motor functions. Its antinociceptive effects are related to the adenosine, but not the opioid, receptor system. Of note, incarvillateine produces neither stimulant nor sedative effect on mice, and does not cause lethality under the antinociceptive doses studied, after acute and even 7 days of treatment, suggesting a low toxicity potential. Therefore, incarvillateine may be a novel leading molecule for development as a non-opioid analgesic.

## Materials and Methods

### Animals

All experimental animals were supplied by Beijing Animal Center (Beijing, China) and maintained on a 12 h light/dark cycle. Male Kunming mice (18–22 g) were used for the writhing test and locomotor activity measurements, and male C57BL/6 mice (8–10 weeks old) were used for other procedures. Animals had free access to food and water. All experimental procedures were conducted in accordance with the guidelines for the use of experimental animals approved by the local ethics committee and the Institutional Review Committee on Animal Care and Use.

### Drugs and reagents

Incarvillateine hydrochlorate was synthesized in-house as previously described^30^, and the purity was greater than 95%. It was dissolved in 1% DMSO in saline and intraperitoneally administered (10 ml/kg body weight). Naloxone, theophylline, DPCPX, DMPX, and SCH58261 were purchased from Sigma-Aldrich (St. Louis, MO, USA). Naloxone and theophylline were dissolved in artificial cerebrospinal fluid (ACSF; 147 mM NaCl, 2.7 mM KCl, 1.2 mM CaCl_2_, 0.85 mM MgCl_2_, 1.0 mM Na_2_HPO_4_, pH 7.4) and i.c.v. administered (5 μl/animal). DPCPX, DMPX, and SCH58261 were dissolved in 1% DMSO in saline and i.p. administered (10 ml/kg body weight).

### Acetic acid writhing test

The antinociceptive effects of incarvillateine were initially assessed using the acetic acid-induced writhing test as described previously^46^. Briefly, mice were i.p. injected with 0.6% acetic acid (0.2 ml), and the number of writhes, characterized by a wave of contraction in the abdomen followed by extension of the hind limbs, was recorded for a period of 15 min, starting at 5 min after injection. Incarvillateine was administered 30 min before acetic acid administration.

### Hot plate test

The hot plate test was performed as described previously^46^. Mice were individually placed on the surface of a hot plate (HUGO SACHS Elektronik-Harvard Apparatus GmbH, March-Hugstetten, Germany) maintained at 55 ± 0.5 °C. The latency was recorded from starting to the end point of jumping, licking, or shaking hind paws. A cutoff time of 60 s was imposed to prevent the possibility of tissue damage. Mice were tested before and repeatedly at different times after incarvillateine administration.

### CFA-induced inflammation and pain

Inflammation and pain were induced over a 48-h period by intraplantar injection of CFA (10 μl, F5881, Sigma-Aldrich) into the left hind paw. Control mice were injected with 10 μl saline. The effects of incarvillateine on hyperalgesia were determined at different times after administration by measuring the thermal sensitivity. To determine the effects of incarvillateine on inflammation, the ventral-dorsal paw thickness was measured with a digital caliper before CFA injection and 30 min after incarvillateine administration, and paw edema rate was calculated as a percentage of change from baseline paw thickness. Mice were then sacrificed, and the injected paws were cut and homogenized in 0.01 M PBS. After centrifugation at 10000 g/min for 5 min, 100 μl supernatant was used for the determination of IL-1β levels by enzyme-linked immunosorbent assay (ELISA).

### SNI-induced neuropathic pain

SNI surgery was performed according to a previously described method^47^ with minor modifications. Briefly, mice were anesthetized with pentobarbital sodium (40 mg/kg, i.p.), and the sciatic nerve and its three terminal branches (the common peroneal, tibial, and sural nerves) were exposed by incising the skin and the biceps femoris muscle on the lateral surface of the thigh. The common peroneal and the tibial nerves were tight-ligated with 5.0 silk and transected distal to the ligation, removing 1–2 mm of the distal nerve stump. During the procedure, any contact or stretching of the sural nerve was avoided. The sham mice underwent the same surgical procedure without ligation or transection. After 5–7 days of recovery, the effects of incarvillateine were determined by measuring mechanical sensitivity with von Frey filaments.

### Paclitaxel-induced neuropathic pain

To induce peripheral neuropathy, mice were injected with paclitaxel (2 mg/kg, i.p.) once daily for 5 consecutive days as described previously^48^. One day after the last paclitaxel administration, the effects of incarvillateine were determined by measuring mechanical sensitivity with von Frey filaments.

### Role of opioid and adenosine receptor systems in incarvillateine antinociception

The possible involvement of opioid and adenosine receptor systems in incarvillateine-induced antinociception was examined in the SNI and CFA models. After recovery from intracerebroventricular catheterization and SNI surgeries, the mice were i.c.v. administered ACSF, the nonselective adenosine antagonist theophylline (10 nmol), or the nonselective opioid antagonist naloxone (10 nmol) 10 min before incarvillateine (20 mg/kg, i.p.) administration. To verify the roles of specific adenosine receptor subtypes, the selective A_1_ adenosine receptor antagonist DPCPX (0.1 mg/kg), the relatively preferential A_2_ adenosine receptor antagonist DMPX (1.0 mg/kg), and the selective A_2A_ adenosine receptor antagonist SCH58261 (0.5 mg/kg) were i.p. administered 15 min before incarvillateine administration. The changes in mechanical or thermal sensitivity were determined 30 min after incarvillateine administration.

### Mechanical sensitivity measurement

Mechanical sensitivity was measured using von Frey filaments according to the up-down method described by Dixon^49^. Mice were individually placed in a plastic enclosure on an elevated wire mesh floor and allowed to habituate for 30 min before testing. A series of von Frey monofilaments were presented to the sural nerve innervation area in the plantar surface of the injured hind-paw, beginning with the 0.6 g filament. The next weaker filament was applied if a brisk paw withdrawal was observed; otherwise, the next stronger filament was applied. After the first cross of response threshold occurred, four additional stimuli were presented. The 50% response threshold was calculated based on the last six stimuli, using the formula by Dixon^49^.

### Thermal sensitivity measurement

Thermal sensitivity was measured with a plantar test device (Model 390 G; IITC Life Science Inc., Woodland Hills, CA, USA) as previously described^50^. Mice were placed in Plexiglas chambers on a heated (30 °C) glass surface and allowed to habituate for 30 min before testing. A mobile radiant heat source was then positioned under the plantar surface of the hind paw and the paw withdrawal latency was recorded. A cutoff time of 20 s was imposed to prevent the possibility of tissue damage. For each time point, each mouse was tested three times with an interval of 2 min. The latencies of the three repetitions were averaged. Testing during grooming or exploratory behavior was avoided^51^.

### Locomotor activity measurement

Mice were allowed to habituate the experimental environment for 30 min before testing. On the testing day, mice were administered vehicle or incarvillateine and placed in Plexiglass boxes (40 × 40 × 35 cm). Animal behavior was recorded for 120 min by a ceiling video camera and analyzed using the EthoVision video tracking system (Noldus, Wageningen, The Netherlands).

### Rotarod test

Rotarod tests were performed by placing mice on a rod rotating at a speed of 15 rpm. Mice were trained once a day for 3 days and those failed to stay on the rod for 3 min were excluded. On the testing day, the time each mouse stayed on the rod was recorded 30 and 90 min after vehicle or incarvillateine administration.

### Statistical analysis

Data are expressed as the mean ± SEM. Multiple comparisons were performed by one-way ANOVA or repeated measures two-way ANOVA followed by Bonferroni *post hoc* test. The level of statistical significance was defined as *p* < 0.05.

## Additional Information

**How to cite this article**: Wang, M.-L. *et al.* Antinociceptive effects of incarvillateine, a monoterpene alkaloid from *Incarvillea sinensis*, and possible involvement of the adenosine system. *Sci. Rep.* **5**, 16107; doi: 10.1038/srep16107 (2015).

## Figures and Tables

**Figure 1:**
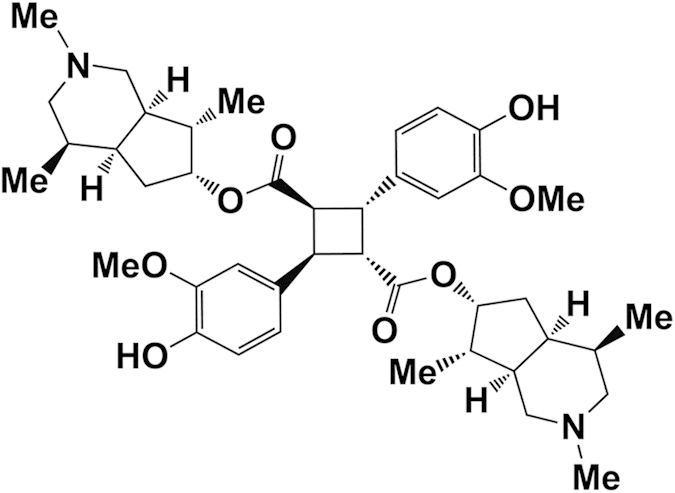
Chemical structure of incarvillateine.
